# Removal of Water-Soluble Extractives Improves the Enzymatic Digestibility of Steam-Pretreated Softwood Barks

**DOI:** 10.1007/s12010-017-2577-2

**Published:** 2017-08-14

**Authors:** Balázs Frankó, Karin Carlqvist, Mats Galbe, Gunnar Lidén, Ola Wallberg

**Affiliations:** Department of Chemical Engineering, Lund University, P.O. Box 124, SE-221 00 Lund, Sweden

**Keywords:** Softwood, Bark, Extractives, Steam pretreatment, Enzymatic saccharification

## Abstract

Softwood bark contains a large amounts of extractives—i.e., soluble lipophilic (such as resin acids) and hydrophilic components (phenolic compounds, stilbenes). The effects of the partial removal of water-soluble extractives before acid-catalyzed steam pretreatment on enzymatic digestibility were assessed for two softwood barks—Norway spruce and Scots pine. A simple hot water extraction step removed more than half of the water-soluble extractives from the barks, which improved the enzymatic digestibility of both steam-pretreated materials. This effect was more pronounced for the spruce than the pine bark, as evidenced by the 30 and 11% glucose yield improvement, respectively, in the enzymatic digestibility. Furthermore, analysis of the chemical composition showed that the acid-insoluble lignin content of the pretreated materials decreased when water-soluble extractives were removed prior to steam pretreatment. This can be explained by a decreased formation of water-insoluble “pseudo-lignin” from water-soluble bark phenolics during the acid-catalyzed pretreatment, which otherwise results in distorted lignin analysis and may also contribute to the impaired enzymatic digestibility of the barks. Thus, this study advocates the removal of extractives as the first step in the processing of bark or bark-rich materials in a sugar platform biorefinery.

**Electronic supplementary material:**

The online version of this article (doi:10.1007/s12010-017-2577-2) contains supplementary material, which is available to authorized users.

## Introduction

Large amounts of bark are produced and are readily available worldwide at sawmills and pulp mills, as bark is removed from the logs during the manufacturing process. In Sweden, 80% of the total standing volume in productive forest lands comprises Norway spruce (*Picea abies*) and Scots pine (*Pinus sylvestris*) species, and an estimated 7.7 million m^3^ of bark is produced annually, based on industrial wood consumption [42]. Today, most bark is combusted at mill sites or district heating plants to produce heat and electricity, although upgrading bark constituents to value-added fuels and chemicals could be beneficial economically and environmentally [30].

Although many of the constituents in wood also occur in bark, the chemical composition and structure of bark differ significantly from those of wood [35]. For example, bark has a lower cellulose and hemicellulose content but typically contains higher amounts of ash, non-cellulosic sugars, and extractives. One of the most disparate compositional characteristic of bark is its large amounts of extractives—i.e., soluble lipophilic (such as resin acids) and hydrophilic components (phenolic compounds, stilbenes) [35]. Extractives from Scots pine and Norway spruce barks have recently been characterized by Bianchi et al. [4, 5], Co et al. [8], Kemppainen et al. [15], Krogell et al. [17], Normand et al. [26], and Vernarecová et al. [48]. Extractives have both traditional (e.g., tannins in the leather industry) as well as a range of new uses—for instance, to produce adhesives, resins, and foams [9, 21]. Certain extractives also have pharmaceutical applications [22, 29].

The emergence of second-generation biofuels has increased the interest in assessing the suitability of softwood barks as a feedstock for renewable fuel production [6, 31, 32, 43]. Unfortunately, the structural complexity and heterogeneity of bark render it more difficult to utilize than wood fractions. Softwoods are generally considered the most recalcitrant type of lignocellulosic feedstock for the production of monomeric sugars by pretreatment and enzymatic hydrolysis [11, 19], but the breakdown of softwood barks to generate monomeric sugars from the carbohydrate part has proved to be even more challenging [10, 52].

The lower holocellulose content of bark inevitably lowers theoretical sugar/ethanol yields; furthermore, extractives can potentially have adverse effects on the biochemical conversion of pretreated material. It has previously been shown that elevated amounts of soluble extractives can impair the hydrolytic performance of the enzymes [16, 50], whereas Kemppainen et al. [14] hypothesized that the condensation reactions of bark extractives during acid-catalyzed steam pretreatment, rendering the otherwise water-soluble extractives insoluble and altering the structure of the solid fraction, results in impaired enzymatic hydrolysis. Thus, more severe pretreatment of spruce bark—through the use of an acid catalyst or higher temperature—resulted in a material that elicited a lower hydrolysis rate and sugar yield when subjected to enzymatic hydrolysis. This finding has negative implications in cases where debarking proves to be technically difficult or uneconomic, but severe pretreatment would also be required to provide reasonable sugar yields (e.g., forest harvest residues). The removal of extractives has mainly been investigated with the idea to valorize the extracted compounds [15, 21], but it also generates a holocellulose-enriched residual and might also improve the enzymatic digestibility [14]. However, the effect of hot water extraction followed by acid-catalyzed steam pretreatment was not examined.

In this study, the effects of hot water extraction of softwood barks on subsequent acid-catalyzed steam pretreatment and enzymatic hydrolysis were assessed. The composition of the non-extracted and the hot water-extracted barks of Norway spruce and Scots pine, as well as the steam-pretreated materials, was analyzed. The enzymatic digestibilities of the barks were determined after steam pretreatment and acid-catalyzed steam pretreatment, with or without prior hot water extraction, to examine the possibility of utilizing water extraction to enhance sugar recovery. The results have implications for bark biorefineries and the pretreatment of softwood forest harvest residues—an abundant raw material that is expected to contain bark.

## Methods

### Raw Materials

The bark of Scots pine, *P. sylvestris*, was obtained from a tree that was sampled from long-term field trials in the Svartbergets experimental forests, Unit of Field-Based Research, Swedish University of Agricultural Sciences (SLU). The bark fraction was separated, chipped to approximately 100 × 10 mm, and stored in plastic bags at −20 °C. The bark of Norway spruce, *P. abies*, was kindly provided by a local sawmill (ATA Timber Widtskövle AB, Everöd, Sweden). The pine and spruce barks were chipped further using a knife mill (Retsch GmbH, Haan, Germany) and sieved to obtain a 2- to 10-mm fraction. Pine bark had a dry matter content of 44 wt%, whereas that of spruce bark was 33 wt%. The raw materials were stored in plastic buckets at 4 °C until use.

### Hot Water Extraction

Water extraction of the raw materials was performed in a 60-L stirred tank in 2 consecutive steps: a 2-h cold water extraction at 6% consistency, followed by a 3-h hot water extraction after the primary extracts were drained and replaced with hot tap water (decreasing the consistency to 5.1%). The conditions of hot water extraction were chosen to facilitate effective removal of water-soluble extractives [15] but to avoid intense hemicellulose removal [17], as well as to provide comparability with previous results on hot water-extracted spruce bark [14, 15, 21]. The temperature was maintained at 25 °C during the cold water extraction, whereas after being heated for 1 h, it was kept at 80 °C for 2 h in the hot water extraction step. The stirring rate (200 rpm) was the same in both steps. More thorough water extraction was performed by repeating the hot water extraction step three times. After extraction, the extracts were drained, and the extracted barks were collected. The extracted materials were filter-pressed at a maximum pressure of 5 bar using a hydraulic press (HP5M, Fischer Maschinenfabrik, Neuss, Germany) to adjust the DM content to 30–35 wt% prior to steam pretreatment.

### Steam Pretreatment

Prior to steam pretreatment, each batch, with a total dry weight of 600 g, was impregnated with gaseous SO_2_ (2.5 wt%, based on the moisture content of raw material) in tightly sealed plastic bags for 20 min at room temperature. Excess SO_2_ was vented before the steam pretreatment by leaving the plastic bags open for 30 min. Steam pretreatment was performed in batches at 210 °C for 5 min in a 10-L reactor, per Palmqvist et al. [28]. Steam pretreatments were also conducted without SO_2_ impregnation at 190 or 210 °C for 5 min. The pretreated slurries were stored at 4 °C prior to subsequent analysis and experiments.

### Enzymatic Hydrolysis

Enzymatic hydrolysis of the pretreated slurry was performed in 2-L Labfors bioreactors (Infors AG, Bottmingen, Switzerland) with a working weight of 1 kg. A water-insoluble solids (WIS) load of 10% mass fraction and Cellic CTec3 enzyme cocktail, kindly provided by Novozymes A/S (Bagsvaerd, Denmark), at a load of 5% mass fraction of WIS, were applied, corresponding approximately to 9 FPU/g WIS. The hydrolysis experiments proceeded for 96 h at 45 °C, with a stirring rate of 400 rpm, at pH 5, maintained with 2.5 M NaOH solution. Samples from the hydrolysis liquid were separated in a centrifuge (Galaxy 16 DH, VWR International, Radnor, PA, USA), Germany) in 2-mL Eppendorf tubes at 16,000x*g* for 8 min. The supernatant was passed through 0.2-μm filters (GVS Filter Technology, Morecambe, UK) and stored at −20 °C. The enzymatic hydrolysis experiments were performed in duplicate.

### Analyses

The total solids content of biomass materials and the total dissolved solids content of liquid samples were determined per the National Renewable Energy Laboratory (NREL) [36]. The WIS content of pretreated slurries was measured using the no-wash method of Weiss et al. [46]. The extractives, structural carbohydrates, lignin, and ash contents of the solid fractions and the composition of the liquid fractions were determined per NREL methods [37–40].

Sugars, organic acids, and other degradation products were quantified by high-performance liquid chromatography (HPLC) on a Shimadzu LC 20AD HPLC system that was equipped with a Shimadzu RID 10A refractive index detector (Shimadzu Corporation, Kyoto, Japan). Samples for sugar analysis were pH-adjusted to 5, if necessary, with CaCO_3_ and centrifuged in 2-mL Eppendorf tubes (16,000×*g* for 5 min). All samples were passed through 0.2-μm filters (GVS Filter Technology) and stored at −20 °C until analysis. Sugars were analyzed on a Bio-Rad Aminex HPX-87P column with a De-Ashing Bio-Rad micro-guard column (Bio-Rad Laboratories, Hercules, CA, USA) at 85 °C using degassed deionized water as the eluent at a flow rate of 0.5 ml/min. Organic acids and other degradation products were analyzed on a Bio-Rad Aminex HPX-87H chromatography column with a Cation-H Bio-Rad micro-guard column at 50 °C, with a mobile phase of 5 mM sulfuric acid at a flow rate of 0.5 mL/min.

### Yield Calculation

The glucose yield in the enzymatic hydrolysis experiments was calculated, based on the total available glucose in the liquid and the solid fraction of the steam-pretreated materials per the following equation. The nomenclature for the equations is presented in Table 1.

$$ {\mathrm{Yield}}_{\mathrm{g}\mathrm{lucose}}=\frac{c_{\mathrm{g}}\times \frac{m}{\uprho_{\mathrm{L}}}\times \left(1-\mathrm{WIS}\right)}{m\times {\mathrm{WIS}}_0\times {\varphi}_{\mathrm{g}\mathrm{lucan}}\times 1.11+{c}_{\mathrm{total}}\times \frac{m_{\mathrm{hydrolysate}}}{\rho_{\mathrm{hydrolysate}}}}=\frac{\mathrm{monomeric}\  \mathrm{glucose}\  \mathrm{after}\ 96\ \mathrm{h}\ \mathrm{enzymatic}\  \mathrm{hydrolysis}\ (g)}{\mathrm{g}\mathrm{lucose}\kern0.5em \mathrm{in}\  \mathrm{the}\  \mathrm{solid}\  \mathrm{phase}+\mathrm{glucose}\ \left(\mathrm{monomeric}+\mathrm{oligmeric}\right)\ \mathrm{in}\  \mathrm{the}\  \mathrm{liquid}\  \mathrm{phase}\  \mathrm{of}\  \mathrm{the}\  \mathrm{pretreated}\  \mathrm{material}\ (g)} $$

**Table 1:** Nomenclature for parameters in the equations

*c*_g_	Glucose concentration (g/L)
${c}_{{\mathrm{g}}_0}$	Initial glucose concentration (g/L)
WIS	Mass fraction of water-insoluble solids (%)
WIS_0_	Initial mass fraction of water-insoluble solids (%)
*m*	Working weight of the reactor (g)
*m*_hydrolysate_	Weight of the liquid fraction of the pretreated material added (g)
ρ_L_	Liquid density (g/L)
*ρ*_hydrolysate_	Liquid density of the liquid fraction of the pretreated material (g/L)
*c*_total_	Total glucose (both monomeric and oligomeric forms) concentration in liquid fraction of the pretreated material (g/L)
*c*_monomeric_	Monomeric glucose concentration in liquid fraction of the pretreated material (g/L)
φ_glucan_	Mass fraction of glucan in the water-insoluble solids of the pretreated material (%)
1.11	Molecular ratio of glucose to glucan (180/162 = 1.11)

The degree of enzymatic hydrolysis was calculated as:

$$ \mathrm{Degree}\  \mathrm{of}\ \mathrm{EH}=\frac{c_{\mathrm{g}}\times \frac{m}{\uprho_{\mathrm{L}}}\times \left(1-\mathrm{WIS}\right)-{c}_{{\mathrm{g}}_0}\times \frac{m_{\mathrm{hydrolysate}}}{\rho_{\mathrm{hydrolysate}}}\times \left(1-{\mathrm{WIS}}_0\right)}{m\times {\mathrm{WIS}}_0\times {\varphi}_{\mathrm{g}\mathrm{lucan}}\times 1.11+\left({c}_{\mathrm{total}}-{c}_{\mathrm{monomeric}}\right)\times \frac{m_{\mathrm{hydrolysate}}}{\rho_{\mathrm{hydrolysate}}}}=\frac{\mathrm{monomeric}\  \mathrm{glucose}\  \mathrm{released}\  \mathrm{during}\  \mathrm{enzymatic}\  \mathrm{hydrolysis}\ (g)}{\mathrm{g}\mathrm{lucose}\  \mathrm{in}\  \mathrm{the}\  \mathrm{solid}\  \mathrm{phase}+\mathrm{oligomeric}\  \mathrm{glucose}\  \mathrm{in}\  \mathrm{the}\  \mathrm{liquid}\  \mathrm{phase}\  \mathrm{of}\  \mathrm{the}\  \mathrm{pretreated}\  \mathrm{material}\ (g)} $$

## Results and Discussion

The digestibility of pretreated softwood barks has been reported to be rather low [10, 51]. One factor that has been suggested to contribute to this is the condensation of water-soluble phenolic compounds during acid-catalyzed steam pretreatment. These compounds remain in the fiber fraction—they are in fact analyzed as acid-insoluble lignin—and can reduce the accessibility to cellulose during enzymatic hydrolysis [14]. As a result, less severe pretreatment (i.e., without acid catalyst) was found to be beneficial for spruce bark. Alternatively, water-soluble extractives can be removed before pretreatment in order to avoid detrimental condensation reactions and enable pretreatment conditions that are sufficiently severe to break down softwood bark. In this study, this step was performed by hot water extraction of softwood barks, after which the extracted materials were subjected to acid-catalyzed steam pretreatment and enzymatic hydrolysis.

### Removal of Water-Soluble Extractives

Hot water extraction was used to remove extractives of spruce and pine barks, and raw material analyses were performed before and after the hot water extraction to determine the total amount removed (Table 2). Spruce bark had a higher total extractives content (24.0%) than pine bark (19.4%), the primary difference between which was the content of water-soluble extractives—the ethanol-soluble extractives content of spruce bark was slightly higher than that of pine bark.

**Table 2:** Composition of raw and the hot water-extracted (HWE) spruce (*Picea abies*) and pine (*Pinus sylvestris*) barks as a percentage of dry matter (% of DM)

Material	Carbohydrates						Lignin		Extractives		Ash
	Glucan	Xylan	Galactan	Arabinan	Mannan	Total	ASL^a^	AIL^b^	Water	Ethanol	
Spruce bark	25.2 ± 0.1	3.8 ± 0.0	2.4 ± 0.0	4.0 ± 0.1	2.3 ± 0.0	37.7	5.5 ± 0.2	24.4 ± 0.2	17.1 ± 0.1	6.9 ± 0.2	2.3 ± 0.1
HWE Spruce bark	27.6 ± 0.6	4.2 ± 0.1	2.6 ± 0.1	4.7 ± 0.2	2.5 ± 0.0	41.6	6.1 ± 0.1	28.1 ± 0.6	7.3 ± 0.5	6.5 ± 0.4	2.7 ± 0.1
3X-HWE Spruce bark^c^	27.2 ± 0.0	5.0 ± 0.0	3.3 ± 0.1	5.1 ± 0.1	2.5 ± 0.0	43.1	6.5 ± 0.1	30.7 ± 0.3	4.7 ± 0.2	6.3 ± 0.1	3.1 ± 0.0
Pine bark	20.0 ± 0.1	4.6 ± 0.0	3.0 ± 0.0	4.1 ± 0.0	3.2 ± 0.1	34.9	4.0 ± 0.1	36.9 ± 0.2	13.2 ± 0.1	6.2 ± 0.1	0.9 ± 0.0
HWE Pine bark	22.0 ± 0.2	5.1 ± 0.0	3.5 ± 0.0	4.9 ± 0.1	3.4 ± 0.1	38.9	4.5 ± 0.1	39.4 ± 0.7	6.4 ± 0.1	6.4 ± 0.2	1.1 ± 0.1

^a^Acid-soluble lignin

^b^Acid-insoluble lignin

^c^Hot water extraction performed three times

Extractives contents between studies should be compared with caution, even for the same species, because they also depend on age, felling season, storage conditions [4, 15], and extraction method [7]. A wide range of extractives content has consequently been reported for spruce and pine barks, ranging from 4.5 to 28.2% for spruce bark [10, 14, 24] versus 3.5 to 19.3% for pine bark [24, 27, 45]. The results of this study are consistent with the extractives content for spruce and pine barks using similar extraction schemes. For spruce bark, Frankó et al. [10] reported 28.2% of total (water- and ethanol-soluble) extractives, whereas Valentín et al. [45] obtained a 13.7% water-soluble extractives content for pine bark.

A major compositional difference between spruce and pine barks that this study noted, apart from the extractives content, was the considerably higher lignin content of pine bark. The total lignin content was 40.9% for pine bark, in contrast to 29.9% for spruce bark. These results are comparable with the reported values for pine (33.7 and 44.9%) and spruce barks (27.9%; 32.8 and 33.8%) [10, 14, 24, 45]. Spruce bark had higher glucan content than pine bark, whereas the contents of the other main carbohydrates were similar between spruce and pine barks. Accordingly, the total content of carbohydrates was higher in spruce versus pine bark. The proportion of C6 carbohydrates to total carbohydrates was nearly the same in both softwood barks (80 and 75%). Similar carbohydrate contents were also reported for spruce and pine barks by Miranda et al. [24]. The ash content was also comparable with the range in the literature [10, 14, 24, 33, 47].

The water extraction scheme removed more than half of the water-soluble extractives from spruce (57%) and pine bark (51%) (Table 2). Consequently, the levels of other bark constituents, such as carbohydrates, lignin, and ash, increased in hot water-extracted barks compared with the non-extracted raw materials. A variety of research approaches and analytical methods have been used to characterize hydrophilic extractives of softwood barks [4, 5, 15, 17, 21]. The extraction yields vary with different factors (e.g., extraction temperature, time, solid loading, particle size, etc.) but water extracts of softwood barks are mainly composed of condensed tannins, stilbene glucosides, and mono- and polysaccharides (e.g., pectic polysaccharides). The chemical composition of water extracts from spruce and pine barks, among other European softwood species, has been analyzed by Bianchi et al. [5]. Although the ratio of condensed tannins relative to total phenolic compounds was high in the water extracts for spruce and pine barks, Bianchi et al. [5] found that the proportion of total phenolic compounds was significantly lower in water extracts from pine bark versus spruce bark (13.0 and 34.1%, respectively).

More thorough water extraction, performed by repeating the hot water extraction 3 times (3X-HWE), removed an additional 15% of the water-soluble extractives from spruce bark, but complete removal of extractives was not achieved. Even though hot water extraction can efficiently remove tannins from bark, condensed tannins cannot be completely extracted due to covalent bonds between the condensed tannins and the cellulose matrix [9, 13].

### Steam Pretreatment

Steam pretreatment with SO_2_ as the acid catalyst is considered a suitable pretreatment method for recalcitrant lignocellulosic feedstocks, such as softwood [12], and was chosen in the current study for the barks. The composition of the water-insoluble solids fractions of the steam-pretreated materials were determined (Table 3). As a result of its lower initial glucan content, the glucan content of steam-pretreated pine barks—non-extracted and hot water-extracted—was considerably lower than in spruce barks. Steam pretreatment removed most of the hemicelluloses in all steam-pretreated materials, but sugars that originated from the hemicellulose, primarily xylose and mannose, were still detected in the solid fraction of the pretreated slurries. No significant difference in holocellulose content was observed between non-extracted and hot water-extracted barks pretreated under the same conditions.

**Table 3:** Composition of water-insoluble fractions of steam-pretreated spruce and pine barks as a percentage of dry matter (% of DM)

Conditions of steam pretreatment	Material	Carbohydrates						Lignin		Ash
		Glucan	Xylan	Galactan	Arabinan	Mannan	Total	ASL^a^	AIL^b^	
210 °C; 5 min; 2.5% SO_2_	Spruce bark	40.4 ± 1.8	1.5 ± 0.2	n.d.	n.d.	0.3 ± 0.0	42.2	2.8 ± 0.2	54.8 ± 0.1	2.4 ± 0.1
	HWE Spruce bark	40.1 ± 0.1	1.3 ± 0.1	n.d.	n.d.	0.2 ± 0.0	41.6	2.5 ± 0.0	53.8 ± 0.2	2.3 ± 0.0
	3X-HWE Spruce bark^c^	41.7 ± 0.0	1.1 ± 0.0	n.d.	0.2 ± 0.0	0.1 ± 0.0	43.1	2.6 ± 0.1	50.9 ± 0.7	2.1 ± 0.0
	Pine bark	26.2 ± 0.0	0.3 ± 0.1	n.d.	0.1 ± 0.1	0.1 ± 0.0	26.7	2.2 ± 0.1	69.3 ± 0.1	0.6 ± 0.0
	HWE Pine bark	27.1 ± 0.2	0.3 ± 0.1	n.d.	0.1 ± 0.1	0.1 ± 0.0	27.6	2.1 ± 0.0	68.3 ± 0.1	0.7 ± 0.1
210 °C; 5 min; No SO_2_	Spruce bark	40.5 ± 0.2	3.4 ± 0.0	0.7 ± 0.0	0.2 ± 0.0	0.5 ± 0.1	45.3	3.4 ± 0.3	48.0 ± 0.6	1.8 ± 0.1
	HWE Spruce bark	42.5 ± 0.5	3.5 ± 0.0	0.5 ± 0.0	0.1 ± 0.0	0.7 ± 0.0	47.3	3.4 ± 0.1	47.8 ± 0.2	1.9 ± 0.0
190 °C; 5 min; No SO_2_	Spruce bark	44.0 ± 0.5	4.2 ± 0.0	0.9 ± 0.0	0.3 ± 0.0	1.4 ± 0.0	50.8	3.4 ± 0.1	47.8 ± 0.2	2.3 ± 0.1

*n.d.* not detected

^a^Acid-soluble lignin

^b^Acid-insoluble lignin

^c^Hot water extraction performed three times

In contrast, the acid-insoluble lignin (AIL) content of the water-insoluble fractions was higher in steam-pretreated barks that were not hot water-extracted, regardless of species (Table 3), although the AIL content was originally lower in the non-extracted raw materials than in hot water-extracted barks (Table 2). The total lignin recovery over steam pretreatment was 116 and 112% for non-extracted spruce and pine barks, respectively, compared with 101 and 107% for the hot water-extracted spruce and pine barks. This difference was most likely due to larger formation of “pseudo-lignin” in the steam pretreatment of non-extracted barks. The lowest total lignin recovery over steam pretreatment (94%) was obtained with 3X-HWE spruce bark. The apparent AIL content of the pretreated materials decreased as more water-soluble phenolic compounds were removed from the barks by hot water extraction prior to steam pretreatment, supporting the hypothesis that water-soluble bark phenolics are rendered insoluble in acid-catalyzed treatments and are subsequently analyzed as insoluble lignin residue [7, 10, 14, 44]. Further, the AIL content was considerably lower for the barks—both non-extracted and hot water-extracted—that were steam-pretreated without the addition of an acid catalyst (i.e., under milder conditions). In the absence of an acid catalyst, the extent of degradation of hemicellulosic sugars during steam pretreatment is lower, which also results in a lower formation of lignin-like compounds (“pseudo-lignin”) [34].

The composition of liquid fractions that were obtained from the steam-pretreated materials (Table 4) did not differ significantly between the non-extracted and hot water-extracted barks, regardless of species. The concentrations of total sugars (expressed in monomeric form) were slightly lower in the liquid fraction of pretreated pine barks than in the corresponding spruce barks; however, the ratios of monomeric and oligomeric sugars were the same for all steam-pretreated materials that were subjected to the same pretreatment conditions—5 to 10% of all dissolved sugars were in oligomeric form after steam pretreatment at 210 °C for 5 min with 2.5% SO_2_. Omitting the acid catalyst in the pretreatment step significantly increased oligomeric sugar levels (55 to 60% of all dissolved sugars). Decreasing the severity of the pretreatment by performing the steam pretreatment at 190 °C shifted the ratio further, with nearly 70% of all dissolved sugars in oligomeric form. Moreover, as a consequence of milder pretreatment conditions, the concentrations of dissolved sugars were slightly lower in the liquid fractions of materials pretreated without the addition of acid catalyst.

**Table 4:** Composition of liquid fractions of steam-pretreated spruce and pine barks

Conditions of steam pretreatment	Material	Total sugars (expressed as monomeric sugar) (g L^−1^)					Inhibitors (g L^−1^)		
		Glucose	Xylose	Galactose	Arabinose	Mannose	HMF^a^	Furfural	Acetic acid
210 °C; 5 min; 2.5% SO_2_	Spruce bark	13.3	4.3	4.7	7.1	5.1	0.6	0.7	1.7
	HWE Spruce bark	13.1	5.4	5.3	8.5	5.3	0.6	0.9	2.0
	3X-HWE Spruce bark^b^	10.2	5.0	5.3	7.8	4.3	0.3	0.6	1.4
	Pine bark	7.3	2.9	5.2	4.7	4.2	1.1	1.1	1.6
	HWE Pine bark	8.1	3.8	6.2	6.4	4.6	1.1	1.0	1.8
210 °C; 5 min; No SO_2_	Spruce bark	6.9	2.2	3.4	4.4	4.9	0.1	0.3	2.2
	HWE Spruce bark	4.8	2.3	2.9	4.1	4.2	0.1	0.1	2.0
190 °C; 5 min; No SO_2_	Spruce bark	6.6	2.4	4.4	8.4	5.4	0.2	0.0	0.7

^a^Hydroxymethylfurfural

^b^Hot water extraction step performed three times

The levels of degradation products (1.4–2.0 g/L acetic acid and 0.3–1.1 g/L HMF and furfural) were similar for hot water-extracted and non-extracted barks pretreated under the same conditions, consistent with earlier studies that found that softwood barks generate less inhibitors during acid-catalyzed steam pretreatment than bark-free softwoods (2–3 g/L acetic acid, 2–6 g/L HMF, and ~ 1.5 g/L furfural) [10, 43]. Steam pretreatment without the addition of an acid catalyst (lower severity) resulted in even lower concentrations of inhibitory compounds (less than 0.3 g/L HMF or furfural) in the liquid fraction of steam-pretreated spruce barks, because the amount of degradation products that are generated during steam pretreatment is a function of the severity of the pretreatment.

### Effects on Enzymatic Digestibility

**Fig. 1:**
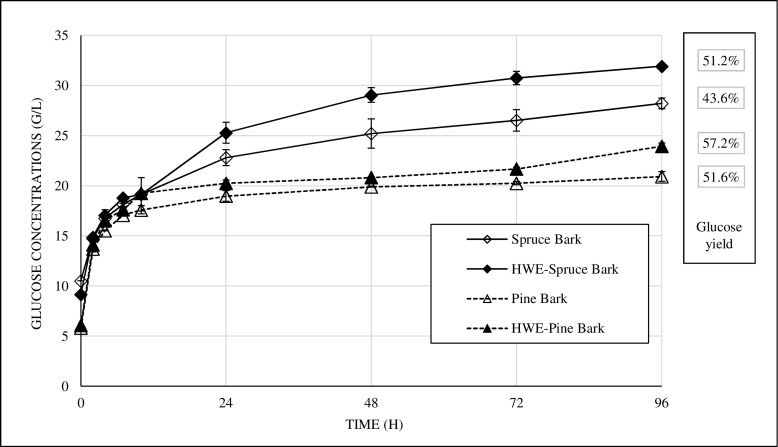
Concentration profiles of glucose during enzymatic hydrolysis and final glucose yields. Enzymatic hydrolysis of spruce (*diamonds*) and pine barks (*triangles*), non-extracted (*open symbols*), and hot water-extracted (HWE) (*filled symbols*), steam-pretreated under the same conditions (210 °C, 5 min, 2.5% SO_2_) at 10% WIS loading, 45 °C, pH 5 for 96 h using Cellic CTec3 enzyme cocktail at a dose of 5 wt% based on WIS. The *error bars* show the lowest and highest concentrations. Total glucose yields expressed as percent of the theoretical value, based on all available glucose in the pretreated materials

**Fig. 2:**
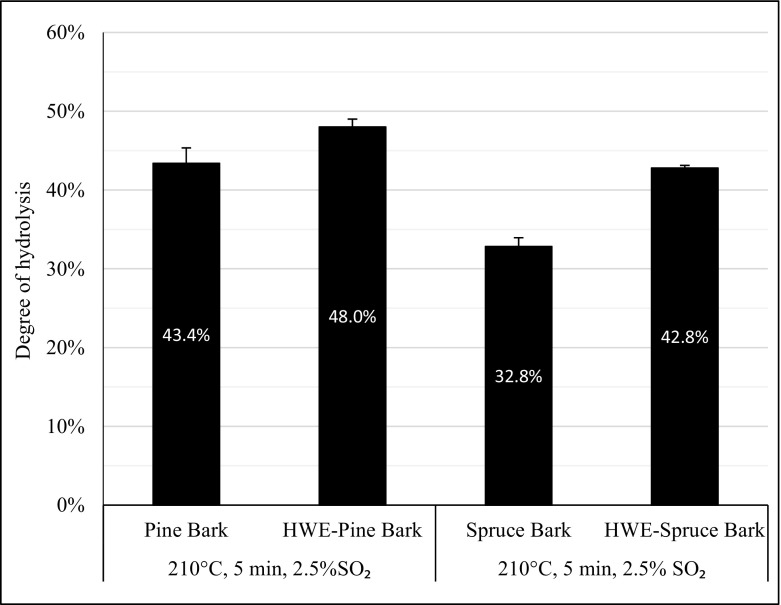
Degree of hydrolysis after 96 h of enzymatic hydrolysis of steam-pretreated pine and spruce barks. Enzymatic hydrolysis of steam-pretreated (210 °C, 5 min, 2.5% SO_2_) pine and spruce barks, non-extracted or hot water-extracted (HWE), at 10 wt% WIS loading, 45 °C, pH 5 for 96 h using Cellic CTec3 enzyme cocktail at a dose of 5 wt% based on WIS. The degree of hydrolysis was calculated based on the sum of oligomeric glucose in the liquid fraction and glucose available in the solid fraction of the steam-pretreated materials. The *error bars* show the lowest and highest values

The glucan content of spruce barks was, as discussed, higher than that of pine barks (Table 3), as was the glucose concentration after enzymatic hydrolysis of spruce barks (Fig. 1). However, the final glucose yields were higher for pine barks than the corresponding spruce barks. The proportion of glucose that was released during steam pretreatment was similar between softwood barks (14.4 and 16.6% for spruce and pine barks, respectively); thus, pine bark showed better digestibility based on the difference in the degree of hydrolysis (32.8 and 43.4% for spruce and pine barks, respectively) (Fig. 2).

In general, softwoods are recalcitrant to biochemical conversion and require high-severity pretreatment conditions [12], high enzyme doses [2], and possibly an additional delignification step [19] to provide a reasonable yield of monomeric sugars. Overcoming the inherent recalcitrance of the bark fractions of spruce and pine has been more challenging for these types of wood fractions [10, 27]. These results are consistent with the glucose yields that were obtained in this study (Fig. 1). For instance, using twice the amount of the same enzyme cocktail, but at the same solids loading as in the current study, the glucose yield was 53% for spruce bark that was pretreated under the same conditions [10]. Higher glucose yields—up to 80%—were reported by Kemppainen et al. [14] for spruce bark but at a significantly lower solids loading (1% dry matter) and an enzyme loading of 25 FPU/g solid Celluclast 1.5 L.

Soluble compounds generated during the pretreatment of softwoods are known to impair microbial fermentation [1] and also the hydrolytic performance of the enzymes. The inhibitory effects of monomeric [49] and oligomeric [20] sugar components and non-sugar components, such as degradation products of sugars, lignin, and extractives [3, 16, 18, 50], have been previously examined. However, decreasing enzymatic digestibility has previously been observed both on whole slurry and on washed fibers with increasing proportions of bark in SO_2_-catalyzed steam-pretreated spruce bark and wood mixtures [10], suggesting that the soluble inhibitory compounds that are liberated during steam pretreatment of bark are not the main cause of the significantly lower enzymatic digestibility of bark versus the wood fraction.

One of the goals of this work was to determine whether enzymatic digestibility can be improved by removing extractives prior to acid-catalyzed pretreatment. Regardless of the species, hot water extraction positively affected the digestibility of the pretreated materials (Fig. 2). However, this favorable effect was more pronounced for spruce bark versus pine bark. The degree of hydrolysis rose from 32.8 to 42.8% and from 43.4 to 48.0% for spruce and pine barks, respectively, from the hot water extraction prior to steam pretreatment. Although barks still remain challenging substrates for enzymatic hydrolysis, this increase in enzymatic digestibility of steam-pretreated spruce and pine barks corresponds to 30 and 11% glucose yield improvement, respectively. The hot water extraction step was more efficient for spruce bark—i.e., a slightly higher proportion of water-soluble extractives was removed. However, because spruce bark originally contained more water-soluble extractives than pine bark, the hot water-extracted barks harbored approximately the same fraction of water-soluble extractives prior to steam pretreatment. This result suggests that there are differences in the chemical structure of the water-soluble extractives fraction of the barks of these softwood species, contributing to the disparate enzymatic digestibilities. Thus, the total amount of remaining water-soluble extractives is not the sole determinant.

**Fig. 3:**
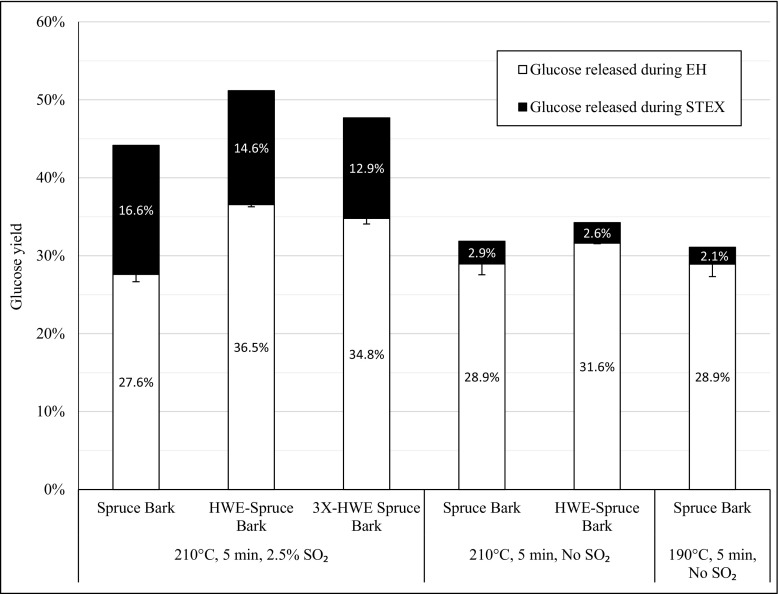
Glucose yield after enzymatic hydrolysis of steam-pretreated spruce barks*.* Enzymatic hydrolysis of spruce barks, non-extracted or hot water-extracted (HWE), steam-pretreated under various conditions at 10% WIS loading, 45 °C, pH 5 for 96 h using Cellic CTec3 enzyme cocktail at a dose of 5 wt% based on WIS. The *filled bars* show the glucose released during steam pretreatment (STEX) step, while the *unfilled bars* represent the glucose released during the enzymatic hydrolysis (EH) step. The *error bars* show the lowest and highest values

Because the effect of hot water extraction on enzymatic digestibility was more prominent with spruce bark and also because its holocellulose content makes it more relevant as a sugar platform than pine bark, additional experiments were performed with spruce bark, including a more extensive hot water extraction (i.e., repeated three times) and steam pretreatments without the addition of SO_2_ (Fig. 3). The steam pretreatment of non-extracted spruce bark at 210 °C for 5 min with 2.5% SO_2_ catalyst, which has been shown to be effective for the pretreatment of spruce wood chips [41], resulted in the lowest yield of glucose that was released during enzymatic hydrolysis. Steam pretreatment without the acid catalyst and a decrease in temperature (lowering the severity of the steam pretreatment) did not significantly improve this yield. These results somewhat contradict a previous study, in which more severe steam pretreatment decreased the rate and yield of hydrolysis [14]. This trend, however, was not seen at higher enzyme doses in that study, and there was no significant difference in the final glucose yields of enzymatic hydrolysis observed after 48 h, regardless of the use of acid catalyst in the pretreatment step. Although the enzyme dose in our experiments was comparable with the low dose in the aforementioned study, the newer, more effective commercial enzyme cocktail that was used in our study might explain the improved, similar enzymatic digestibility, regardless of the addition of acid catalyst or the decrease in temperature in the steam pretreatment.

However, with regard to total glucose yields (Fig. 3), it is apparent that the use of an acid catalyst during the steam pretreatment was highly beneficial when the monomeric glucose that was released during the steam pretreatment was included. Total glucose yield of 31.9% was obtained after enzymatic hydrolysis of non-extracted spruce bark that was steam-pretreated for 5 min without acid catalyst at 210 °C, whereas addition of the acid catalyst increased the total glucose yield to 43.6%. When comparing hydrolysis data with the results of Kemppainen et al. [14], it should be noted that the acid catalyst and the impregnation method differed in the former study (soaking in 0.5% sulfuric acid solution), which might also have contributed to the difference in total glucose yields. Nevertheless, the total amount of monomeric glucose that was liberated from non-extracted spruce bark by steam pretreatment and enzymatic hydrolysis was considerably higher when acid catalyst was used in the pretreatment step in the present study.

A detailed analysis of interactions between extractives that have been isolated from various wood fractions and cellulose surfaces has previously shown that deposition of the phenolic extractives fraction from pine wood on microcrystalline cellulose negatively affected the glucose release during enzymatic hydrolysis [23]. The partial removal of water-soluble extractives by hot water extraction before the steam pretreatment step improved the enzymatic digestibility of spruce bark. The degree of enzymatic hydrolysis and total glucose yields were greater with hot water-extracted spruce bark in all cases, but the positive effect was significantly better when the steam pretreatment was performed with an acid catalyst (32 and 9% improvement in the degree of hydrolysis with and without an acid catalyst in the pretreatment step, respectively). This result is consistent with the explanation that water-soluble extractives undergo detrimental changes during steam pretreatment that impair the subsequent enzymatic hydrolysis, especially when steam pretreatment is performed in the presence of acid catalyst. Despite the improvements in the enzymatic digestibility of both barks by hot water extraction prior to pretreatment, the total glucose yields remained lower than previous results on the stem wood fraction of spruce [10, 25]. Additionally, a more thorough hot water extraction step, resulting in the removal of an additional 15% of water-soluble extractives before the acid-catalyzed steam pretreatment, did not result in further improvements in the degree of hydrolysis or total glucose yield (Fig. 3). Clearly, bark remains a challenging substrate for enzymatic hydrolysis.

## Conclusions

The use of acid catalyst during steam pretreatment was found to be beneficial in reducing the recalcitrance of softwood barks from spruce and pine. However, the formation of water-insoluble “pseudo-lignin” from water-soluble bark extractives during acid-catalyzed steam pretreatment resulted in distorted lignin analysis of the pretreated materials and potentially contributed to an impaired enzymatic digestibility. The acid-insoluble lignin content of the pretreated materials decreased as more water-soluble phenolic compounds were removed from the barks by hot water extraction prior to steam pretreatment, whereas no significant difference in holocellulose content was observed between non-extracted and hot water-extracted barks pretreated under the same conditions. Partial removal of water-soluble extractives by hot water extraction improved the enzymatic digestibility of steam-pretreated softwood barks. The obtained increase in enzymatic digestibility of steam-pretreated spruce and pine barks after extraction corresponded to 30 and 11% glucose yield improvement, respectively.

## Electronic supplementary material

ESM 1 (PDF 87 kb)

## Abbreviations

AIL: acid-insoluble lignin
ASL: acid-soluble lignin
DM: dry matter
EH: enzymatic hydrolysis
FPU: filter paper unit
HPLC: high-performance liquid chromatography
HWE: hot water extracted
NREL: National Renewable Energy Laboratory
STEX: steam explosion
WIS: water-insoluble solids
