# Supplementary material for: Removal of Water-Soluble Extractives Improves the Enzymatic Digestibility of Steam-Pretreated Softwood Barks
Source: Appl Biochem Biotechnol. 2017 Aug 14;184(2):599–615. doi: 10.1007/s12010-017-2577-2 (PMC5767193; doi:10.1007/s12010-017-2577-2)
Supplement: Supplementary file 1 — (PDF 87 kb) [file 12010_2017_2577_MOESM1_ESM.pdf]

Online Resource 1 *Results of enzymatic hydrolysis experiments*

Enzymatic hydrolysis of pine and spruce barks, non-extracted or hot water-extracted (HWE), steam-pretreated under various conditions at 10 wt-% WIS loading, 45°C, pH 5 for 96 h using Cellic CTec3 enzyme cocktail at a dose of 5 wt-% based on WIS.

Conditions of steam pretreatment	Material	Yield of glucose released during steam pretreatment (%)[1]	Yield of glucose released during enzymatic hydrolysis (%)[1]	Total glucose yield (%)[1]	Degree of hydrolysis (%)[2]	Final glucose concentration (g/L)
210°C; 5 minutes; 2.5% SO_2	Spruce Bark	16.6	27.7	43.6±0.9	32.8±1.1	28.2±0.5
	HWE Spruce Bark	14.6	36.5	51.2±0.3	42.8±0.3	31.9±0.1
	3X-HWE Spruce Bark[3]	12.9	34.8	47.7±0.7	40.4±0.8	30.2±0.4
	Pine Bark	14.4	37.1	51.6±1.7	43.4±2.0	20.9±0.5
	HWE Pine Bark	14.7	42.6	57.2±0.9	48.0±1.0	24.0±0.3
210°C; 5 minutes; No SO_2	Spruce Bark	2.9	28.9	31.9±1.4	29.8±1.4	18.3±0.7
	HWE Spruce Bark	2.6	31.6	34.2±0.1	32.5±0.1	19.7±0.0
190°C; 5 minutes; No SO_2	Spruce Bark	2.1	28.9	31.1±1.6	29.6±1.7	19.0±0.7

[1] The glucose yield in the enzymatic hydrolysis experiments was calculated on the basis of total available glucose in the liquid and solid fractions of the steam-pretreated materials

[2] The degree of hydrolysis in the enzymatic hydrolysis experiments was calculated based on the oligomeric glucose in the liquid fraction and glucose available in the solid fraction of the steam-pretreated materials

[3] Hot water extraction performed 3 times
